# Use of Dietary Supplements and Perceived Knowledge among Adults Living with Fibromyalgia in Norway: A Cross-Sectional Study

**DOI:** 10.3390/nu14010005

**Published:** 2021-12-21

**Authors:** Linda Aimée Hartford Kvæl, Ida Løchting, Marianne Molin

**Affiliations:** 1 Norwegian Social Research—NOVA, Department of Ageing Research and Housing Studies, Oslo Metropolitan University, P.O. Box 4 St. Olavs Plass, N-0130 Oslo, Norway; 2 Department of Physical Therapy, Faculty of Health Sciences, Oslo Metropolitan University, P.O. Box 4 St. Olavs Plass, N-0130 Oslo, Norway; 3 Norwegian League Against Rheumatism, Professor Dahls Gate 32, N-0260 Oslo, Norway; ida@revmatiker.no; 4 Research and Communication Unit for Musculoskeletal Health (FORMI), Division of Clinical Neuroscience, Oslo University Hospital, P.O. Box 4956, N-0424 Oslo, Norway; 5 Department of Nursing and Health Promotion, Faculty of Health Sciences, Oslo Metropolitan University, P.O. Box 4 St. Olavs Plass, N-0130 Oslo, Norway; mmolin@oslomet.no; 6 Department of Health, Oslo New University College, Lovisenberggata 13, N-0456 Oslo, Norway

**Keywords:** fibromyalgia syndrome, dietary supplements, musculoskeletal disorders, health claims, informed health choices, Norway

## Abstract

Fibromyalgia syndrome (FMS) is a complex medical condition characterized by widespread musculoskeletal pain. To date, no gold standard treatment has been developed, and persons with FMS often seek alternative methods to control their symptoms, such as dietary supplements (DS). This study aimed to describe the use of DS in persons living with FMS and examine the associations between the use of DS and its potential predictors. We recruited a convenience sample of 504 participants (≥18 years) living with FMS. The main outcome variables included estimated expenditure on DS in the last 12 months in Norwegian kroner (NOK) and the differences between the groups of users and non-users of DS. Of the 504 participants, 430 reported having used DS, and the mean amount of money spent in the previous year was determined to be NOK 2300. The most common DS reported were vitamin D, magnesium, and omega-3 fatty acids. The predictors of being a DS user were high education, high self-reported knowledge of DS but low overall knowledge of health claims. Users of DS marketed for muscles/joints appear to spend more money on DS. The increasing availability of DS and aggressive advertising in the media through health claims stipulate the need for interventions that lead to informed decisions about DS.

## 1. Introduction

Fibromyalgia syndrome (FMS) is a complex medical condition of unknown etiology characterized by widespread musculoskeletal pain accompanied by fatigue, joint stiffness, depression, anxiety, disturbed sleep, as well as gastrointestinal and cognitive challenges [1,2,3,4]. FMS is now considered to be one of the most usual chronic pain syndromes and recognized as the second most common condition in rheumatology after osteoarthritis [5]. The worldwide mean prevalence of FMS has been estimated to be 1.78%, and the condition is more common among women than men [6]. In addition to the female gender, FMS is associated with higher age, low levels of education, low socioeconomic status, and living in rural districts [7]. To date, no gold standard treatment for FMS exists, and typically, persons with FMS receive a combination of pharmacotherapy, physical therapy, and cognitive behavioral therapy [8]. However, FMS patients seldom achieve full remission concerning their condition, where at best 25% report long-term effects [9]. Hence, persons with FMS often seek alternative methods to control their symptoms [10].

Recently, the literature has shown an increasing interest in nutritional interventions as well as in the use of dietary supplements (DS) among persons living with FMS [11,12,13]. DS can be defined “as a product taken orally that contains one or more ingredients that are intended to supplement one’s diet and are not considered food” [14]. FMS results in psychological burden and high economic costs, both at the individual and at the social levels [15], and persons living with FMS might be more willing to use DS [16]. Arranz et al. (2012) revealed in their survey that 73% of the persons living with FMS were DS users, and 61% of those persons became users after the onset of the disease [17]. Consequently, compared to the normal population, there seems to be an association between being affected by FMS and higher use of DS [18].

DS are condensed sources of nutrients or similar substances with nutritional or physiological benefits used to supplement a normal diet; they were produced as pills, capsules, or liquids in measured doses [19]. However, it must be noted that high doses of some vitamins, especially when taken regularly, can be toxic [20]. Therefore, uncritical use of DS could be a risk rather than a benefit for the health of potential users, such as those individuals living with FMS or other rheumatic and/or musculoskeletal disorders. Therefore, an accurate understanding of DS appears to be a prerequisite for making informed DS choices, especially considering the fact that health claims in the media may provide an uncritically positive image of DS that shapes DS intake patterns [21].

Regarding food interventions for the management of FMS symptoms, a relatively new systematic review reported that persons with FMS achieved remarkable pain relief by following a FODMAP or vegan diet and consuming DS with *Chlorella* green algae, acetyl-l-carnitine, coenzyme Q10, or a mixture of vitamins E and C [12]. Similarly, Silva et al. (2019) found in their systematic review that pain and functional consequences in FMS persons seem to improve when a raw vegetarian diet, a low FODMAP diet, or a hypocaloric diet is followed, in addition to improvement in anxiety, depression, sleep quality, inflammatory biomarkers, and, consequently, the overall quality of life [11]. Nevertheless, Paglia et al. (2020) underline that even though the research literature indicates that DS with vitamin D, iron, probiotics, and magnesium show somewhat optimistic results in clinical trials, the role of DS remains controversial [13]. Nevertheless, from the abovementioned reviews, the general conclusion is that the overall strength of these studies is weak as a result of poor study design, wide study heterogeneity, small sample sizes, and high degrees of bias [11,12,13]. Hence, the knowledge presented is inadequate in suggesting a specific nutritional intervention or use of DS for the management of FMS, and as such, further research is necessary [11,12,13].

Overall, there is an urgent need for more knowledge on the use of DS in FMS management and its crucial determinants that shape the usage patterns to make the best evidence-based recommendations for future disease management. Currently, few studies have examined the consumption of DS among patient groups in Norway, including persons with FMS. Therefore, this study aims to describe the use of DS in persons with FMS in the country and investigate the associations between the use of DS and its potential determinants, including demographics, lifestyle choices, health conditions, and general knowledge of DS. Based on the current knowledge of the use of DS in the common population, we hypothesized that within a group of people with FMS; (1) the estimated money spent on DS is higher compared to the normal population; (2) the self-perceived knowledge about DS is higher than the actual knowledge; and (3) the predictors for use are higher age, higher education, good self-perceived health, and better lifestyle.

## 2. Materials and Methods

### 2.1. Study Design

The study is cross-sectional, and for this study, data were collected from a survey conducted among the members of a national user organization in Norway.

### 2.2. Study Population and Recruitment

The analysis involved a convenience sample of participants aged ≥ 18 living with a musculoskeletal disorder that are registered members of the included user organization. The organization is a national volunteer-driven organization established by a group of persons living with FMS. Its objective is to deliver a forum for the exchange of knowledge and experiences of persons affected by FMS, as well as to promote their voices at the national level. Invitations for participation were sent via e-mail as a newsletter. The newsletter provided information pertaining to the purpose of the study and included a link to the online survey. Data collection took place from 11 December 2020 to 13 January 2021; during this period, the link was accessible. At the time of the survey, the number of registered members of the organization was 5825 (1 January 2021).

### 2.3. Survey

The online anonymous survey was developed from June to October 2020. The project group consisted of researchers with experience in survey methodology and clinical knowledge in rheumatic and musculoskeletal disorders (RMDs), a representative from a user organization (the Norwegian League Against Rheumatism), as well as persons living with RMDs. The survey was developed in Nettskjema, a net-based instrument for developing and conducting online surveys, which is organized by the University Information Technology Center at the University of Oslo, Norway. Survey development had three phases. First, the survey was tested and evaluated by professionals and experts in the field of RMDs and survey methodology. Then, the survey was piloted with 14 RMD patients and finally retested on three experts and two persons with RMDs who had participated in the previous rounds. Adjustments were made to survey length, technical errors, question formulation, wording, diagnoses, and user friendliness. The final survey that formed the foundation of this article consisted of three parts: (1) informed consent, (2) demographics, lifestyle factors, and health conditions, and (3) the use and knowledge of DS.

### 2.4. Variables

#### 2.4.1. Outcome Variables

The main outcome variables were total expenditure on DS in the last 12 months in NOK and DS use/non-use within the past 12 months and the last week. For the DS use/non-use variable, the survey participants were shown a list of 32 DS, some of which were specifically targeted towards musculoskeletal disorders, while others were selected based on DS use collected in nutritional studies, including NORKOST 3 [22], NAFKAM 2018 [23], and the Norwegian Mother, Father, and Child Cohort Study [24]. Further, besides the predefined list of 32 DS, the option “other” allowed the participants to report any other DS not included in the list. The users of DS were further categorized into five groups: (1) omega-3 fatty acids or fish oil users, (2) multivitamin or -mineral users, (3) single-vitamin or -mineral users, (4) non-vitamin or -mineral users, and (5) users of the DS marketed specifically for RMDs.

#### 2.4.2. Covariates

Demographics, number of diagnoses, lifestyle factors, health conditions, and knowledge of DS were recognized as potential covariates in the multivariate models. The demographic variables included age, gender, education, employment, marital status, health professional background, height, and weight. Lifestyle factors included smoking status, alcohol use, snuff habits, BMI, exercise, and consumption of fruits and vegetables. Drawing from Kofoed et al. (2015), we made a health matrix in line with Norwegian nutritional and lifestyle recommendations, including these five lifestyle factors with a scoring system from 0 to 5 [25].

Health conditions included the number and names of diagnoses besides FMS, the number of medications (high/low), and self-reported health. Self-reported health in general was assessed on a six-point Likert scale, while self-reported health for today was described using a VAS ranging from 0 to 100, where 100 denotes the best health imaginable and 0 the worst [26]. To assess the participants’ knowledge of DS, they were asked to score their knowledge of DS on a six-point Likert scale. In addition, they were asked if they had informed/asked their doctor for advice regarding the use of DS. The participants were also asked to report which source(s) they used to gain knowledge of DS including GPs, other healthcare professionals, and pharmacists. Finally, inspired by Karbownik et al. (2021), a matrix was developed with various health claims about DS [21]. For this, the respondents were asked to what extent they agreed or disagreed with nine various health claims using a six-point Likert scale. Total scores were attained for summing up the scores for each variable, ranging between 9 and 54.

### 2.5. Statistical Analysis

Statistical analyses were managed using the Statistical Package for the Social Science (SPSS) version 27. Descriptive data were illustrated with the mean and standard deviation (SD) values for interval data and percentages and proportions for categorical data. To assess statistical differences between the groups, in this case, the users and non-users of DS, the *t*-test and the chi-squared test were conducted for the interval data and the categorical data, respectively.

To explore bivariate and multivariate associations between the estimated amount of money (NOK) spent the previous year on DS and the independent variables, we conducted linear regression analyses. To ensure that the assumptions for linear regression analysis were present, each of the univariate regression models was inspected [27]. From the unadjusted linear regression analyses, we included only the variables with significant associations with the outcome in the multiple linear regression model in addition to gender and age. To consider the strength of the associations between the different potential predictors and money spent on DS in the previous year, we used the standardized betas from the linear regression models with their p-values and the adjusted coefficient of determination (R^2^).

Furthermore, logistic regression analyses were applied to explore potential predictors of being in the group of DS users vs. being in the group of non-users. Similarly, the variables showing the strongest associations in the unadjusted analyses were included in the multivariate logistic regression model in addition to age and gender. Based on the logistic regression analyses, the odds ratio (OR) illustrated the strength of association between the users and non-users of DS and the independent variables. We decided the level of statistical significance to be *p* < 0.05 in all the analyses.

## 3. Results

### 3.1. Sample Characteristics

A total of 513 participants answered the survey; however, two of them decided not to participate, one did not have an RMD, one was under examination, and five had conditions other than FMS. Therefore, the final number of participants was 504, which gave us a response rate of 8.65%.

The characteristics of the whole sample and the users/non-users of DS are illustrated in Table 1. Of the 504 participants included in the study, 94.4% were women with a mean age of 52.7. All the participants were suffering from FMS (*n* = 504); however, over 60% had one or more additional diagnoses (mean = 2.2; SD = 1.3). The most common additional diagnoses were osteoarthritis (*n* = 162), lower back pain (*n* = 138), or chronic neck pain (*n* = 133). Only a few had rheumatoid arthritis (2%) and Bechterew’s disease (0.6%) in addition to FMS. Over 80% reported taking regular medications, and out of these, 45.6% used more than two medicines. About 76% of the participants were living with a partner, and overall, two out of five were employed when the survey was carried out. Most of the participants were born in Norway (*n* = 469), and one in four participants had a health professional background. The participants came from all parts of Norway, with a majority hailing from the southeast of Norway. There was no statistically significant difference between users and non-users of DS in terms of self-reported health in general and self-reported health today. Overall, 85.3% of the participants reported that they had used DS in the previous year. The users of DS had a significantly higher education than the non-users (*p* = 0.01). Furthermore, the users of DS rated their knowledge of DS to be higher than the non-users (*p* = 0.01) and reported using reliable sources of knowledge (*p* = 0.01). However, in terms of scoring the health claims in the online survey, they revealed significantly lower scores than the non-users (*p* = 0.02), indicating lower overall knowledge of DS among users.

### 3.2. Prevalence of DS

Out of the 504 participants, 430 (85.3%) reported having used DS, spending the mean amount of NOK 2300 (range, 0–35,000) in the previous year. The five most common reasons for using DS were to treat or prevent a chronic health problem (37.7%), to follow doctor’s recommendation (37.1%), to get more energy (34.5%), to reduce inflammation in the body (33.9%), and to ensure adequate nutrient uptake (30.2%). The least common reasons for the use of DS were to lose weight (3.4%) or to get nicer skin (2.4%). Among the group of users, a total of 328 participants (76.3%) reported that they had consumed vitamin D supplements, 279 participants (64.5%) took magnesium, and 265 (61.6%) had omega-3 fatty acids in the last year. These three types of DS were also the most frequently consumed on a weekly basis. Types of DS used least frequently were St. John’s wort (2.1%), conjugated linoleic acid (CLA) (2.6%), and valerian (2.6%). Table 2 shows the frequency and percentage of the use of types of DS. The users of DS were further grouped into five user profiles: (1) omega-3 fatty acids or fish oil users, (2) multivitamin or -mineral users, (3) single-vitamin or -mineral users, (4) non-vitamin or -mineral users, and (5) users of the DS marketed specifically for RMDs.

### 3.3. Lifestyle Factor and Health Conditions

Regarding lifestyle factors, we used five variables, namely, smoking status, snuff habits, alcohol use, physical activity, and consumption of fruits and vegetables. In general, Table 3 illustrates that the use of cigarettes and snuff was low among most of the participants, although 11.3% reported regular smoking. Approximately 40% consumed alcohol 1–4 days a week, and only 24.2% engaged in physical activities (5–7 days/week). More than 60% said that they did not eat enough fruits and vegetables (less than 5 days a week). When expressing lifestyle factors as the health index score from 0 to 5 where a higher number indicates a better lifestyle, the mean score of the whole sample was found to be 3.36 (SD = 0.85).

Regarding self-reported health in general, the distribution of scores was as follows: very bad (1.8%), bad (37.9%), quite bad (12.3%), quite good (38.3%), good (9.5%), and very good (0.4%), with a mean score of 3.2 points (SD = 1.1). The VAS scale measuring self-reported health today had a mean score of 50.3 for a range of 0–90 (SD = 17.6).

### 3.4. Knowledge of DS

**Figure 1:**
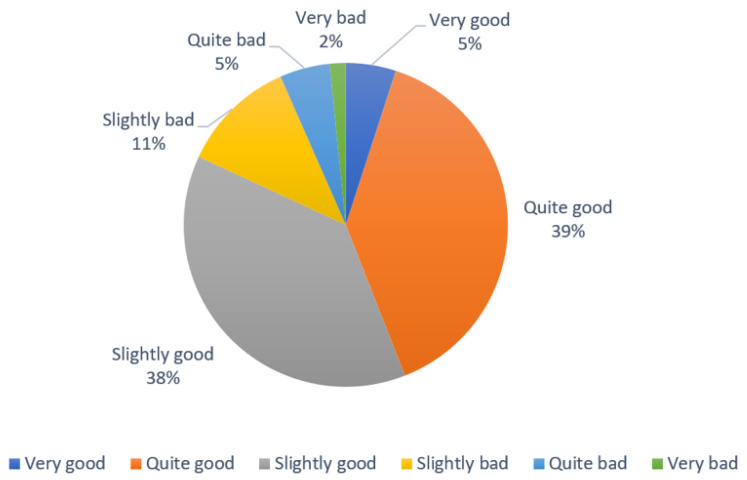
Self-reported knowledge of DS in percentage (%).

The participants reported their perception of the extent of their knowledge of DS on a scale of very good, quite good, slightly good, slightly bad, quite bad, and very bad. Figure 1 shows that most of the participants (82%) perceived their knowledge of DS as slightly good, quite good, and very good, while 18% reported their status of knowledge as rather poor.

Furthermore, to determine the participants’ current knowledge of DS, they were asked to answer whether they agreed or disagreed with nine different health claims [21]. Table 4 shows the frequency of correct answers for the health claims based on the current evidence. The claim “The use of DS may influence the effect of medicines” had the highest number of correct answers (77%), while the claim “DS must undergo tests to ensure that they are safe before they can be sold on the market” had only 18 correct answers, indicating that 96.4% answered this claim incorrectly.

From the possible range of 9–54 where a higher score indicates better knowledge of DS, the lowest score in the group was 10 and the highest was 48. The final sum score expressing the overall knowledge of DS assessed through the abovementioned nine claims had a mean score of 26.9 (SD = 6.2).

### 3.5. Predictors of Spending Money on DS

The unadjusted linear regression models (Table 5) highlighted significant associations between money spent in the last year on DS and background characteristics such as education (*p* = 0.009) and marital status (*p* = 0.04), implying that higher education and not having a partner are associated with more money spent on DS. Furthermore, the unadjusted analyses revealed a significant relationship between the money spent in the last year on DS and the variables self-reported knowledge about DS (*p* = 0.02) and the knowledge score of health claims (*p* = 0.001). Accordingly, although the participants who spent more money on DS perceived their knowledge as high, they had an overall lower score on the knowledge of health claims. In addition, the unadjusted linear regression analyses revealed significant associations between the money spent on DS in the last year and the three subgroups of non-vitamin or -mineral users of DS (*p* = 0.03), users of the DS marketed for muscles/joints (*p* = 0.001), and omega-3 fatty acids or fish oil users of DS (*p* = 0.02), suggesting that these three profiles of users spent more money on DS.

However, in the adjusted linear regression analysis (Table 6), the variables still showing a significant relationship between money spent on DS in the last year were education (*p* = 0.02), knowledge of health claims (*p* = 0.001), and users of DS marketed for muscles/joints (*p* = 0.01), as well as a statistical trend towards not having a partner (*p* = 0.06). To summarize, although the participants spending more money on DS had higher education, they gave more wrong answers on health claims, indicating an overall lower knowledge of DS. In addition, users of supplements marketed for muscles and/or joints spent more money on DS in the last year.

### 3.6. Predictors of Being in the Group of Users

The unadjusted and adjusted logistic regression models (Table 7) showed significant differences between the users (*n* = 430) and non-users of DS (*n* = 74) in terms of the variables measuring education (*p* = 0.003), self-reported knowledge of DS (*p* = 0.001), knowledge of health claims (*p* = 0.004), and a statistical trend towards the use of reliable sources of knowledge of users of DS (*p* = 0.09). This implies that the predictors of being in the group of DS users are high education, high self-reported knowledge of DS but rather low overall knowledge of health claims. A one-unit increase in sum score expressing the knowledge of health claims decreased OD by 6%, moving from low to high education increased OD by 141%, while one-unit increase in self-reported knowledge of DS increased OD by 81% for being in the group of users of DS adjusted for gender and age.

### 3.7. Summary of the Main Findings

Among the 504 participants with FMS, 430 reported having used DS where the mean amount of money spent the last year was NOK 2300. Vitamin D, magnesium, and omega-3 fatty acids were the most commonly used DS. The predictors of being in the group of DS users are high education, high self-reported knowledge of DS but rather low overall knowledge of health claims. Users of the DS marketed for muscles/joints spent more money on DS.

## 4. Discussion

This article aimed to describe the use of DS in persons with FMS, as well as to examine the associations between the use of DS in this population and the important determinants such as demographics, lifestyle factors, health conditions, and general knowledge of DS. Of the included participants, over 90% were women with a mean age of 52.7 years. Although our study included a higher number of women, this is consistent with the fact that FMS is more usual among women than men, with a proportion of 9:1 [28]. Previous research also shows that DS use increases with age and is more prominent in women than men [29,30].

Our results indicated that a large proportion of people with FMS used DS. According to the latest nationwide diet survey NORKOST 3, 53% of women and men use DS [22]. In a 2015 study with the members of the National Association for Heart and Lung Disease (LHL) aged 50 or older, 81% reported regularly using DS [31], and among Norwegian middle-aged women with cancer, 71% reported to have used DS in the last week [32]. Hence, DS use among people with FMS seems to be more widespread compared to the general population and possibly also compared to people with other diagnoses [33]. The amount of money spent on DS reported in our study was also higher compared to the NAFKAM study, which illustrated that Norwegians spend approximately NOK 1000 annually on DS [23]. Within our results, vitamin D was the most commonly used DS. In a recent consumer survey conducted in the US, vitamin D was the second most popular DS, largely in the 55+ age group with nearly 50% of DS users [34]. However, according to Martins et al. (2019), the findings on the prevalence of hypovitaminosis D in the FMS population are rather inconclusive. Karras et al. (2016) highlighted that although vitamin D is recommended in persons with a high risk of developing vitamin D deficiency or those diagnosed with hypovitaminosis D, DS containing vitamin D still cannot be recommended in FMS persons on a daily basis [35].

The most common reasons reported by the participants for DS use are to treat or prevent a chronic health problem, to follow doctor’s recommendations, to get more daily energy, to reduce inflammation in the body, and to ensure adequate nutrient uptake. These reasons correspond well with the findings of similar research that common motivations for the use of DS are to prevent disease, to enhance mental and general health, to enhance sports performance (energy), and to compensate for dietary deficiencies [36,37]. Furthermore, the group of DS users in our study showed a trend towards the use of reliable information sources including doctors, healthcare professionals, or pharmacists. This is in line with Dickinson et al. (2015) where 82% of the survey respondents agreed that persons considering taking high doses of DS should consult with their doctor [38].

A few interesting patterns in our results were that the users of DS had significantly higher education and their perceived knowledge of DS was significantly higher, and also they used reliable information sources. However, this group simultaneously scored significantly lower in their knowledge of health claims and overall knowledge of DS compared to the non-user group. The fact that users of DS often have higher education is supported in the literature [39,40]. In addition, according to Bailey et al. (2013), DS users often report better health, make rather cautious use of alcohol, do not smoke, and exercise more frequently than non-users [40]. This is also in line with the report of a series of surveys (2014) that found that users of DS were, to a large extent, more likely than non-users to claim they try to eat a balanced diet, visit their doctor regularly, exercise more often, and maintain an appropriate weight [29]. Accordingly, it seems that DS users are more concerned about a healthy lifestyle; however, in line with our findings, they might also think they know more about DS than they actually do. This phenomenon is also known as cognitive bias called the Dunning–Kruger effect [41]. Therefore, the increasing availability of DS, aggressive advertising in the media making health claims, and the frequent ideas that DS is good for general health call for more knowledge regarding these patterns [42].

Interestingly, our results showed no statistically significant difference between users and non-users of DS in terms of self-reported health in general and self-reported health today. This is in line with the research literature which indicates that DS does not affect the health of patients with fibromyalgia [11,12,13]. Although this association can be interpreted as an indication that the use of DS has no palliative effect, this is a result that must be further investigated using other study designs that are suitable for saying something about causality, such as randomized controlled trials.

Users of the DS marketed for muscles/joints seem to spend more money on DS. Accordingly, persons with RMDs, including FMS, are more willing to use alternative treatments such as DS to improve or treat their chronic disease [16]. Therefore, interventions fostering informed decisions regarding DS seem necessary. Our results support findings from a recent national survey in Norway from 2021 assessing health literacy, revealing that 33% of the population seem to lack key skills to acquire, understand, and use health information. In addition, the report illustrates that patients with long-term illnesses, such as FMS, may have weaker skills than others and thus may have challenges in understanding information about the illness. Furthermore, around half of the population experience difficulties in assessing whether health information provided by the mass media is reliable. Hence, the report calls for measures to develop the population’s health literacy in general and that health services adapt the health information provided to a greater extent [43]. Our study supports this conclusion.

### Strengths and Limitations of the Study

Cross-sectional studies require data picturing a specific point in time and are suitable for assessing the prevalence and patterns of a large population at little or no expense [44]. A cross-sectional design, as we have used in this study, literally describes the phenomenon of interest and observed associations. However, an apparent consequence is that we cannot provide significant evidence about the direction of the cause-and-effect association [45]. We know that FMS is more prevalent among women and individuals aged higher [6,7], and there is also a pattern of comorbidities among patients with FMS [46]. A review reveals that among adult FMS patients in Norway, approximately 90% are women [47], and most patients are in their 40s and 50s [48]. This is in accordance with our study sample where 94.4% were women with a mean age of 52.7 years (age range of 21–81). In addition, all the participants in our study were suffering from FMS; however, over 60% had one or more additional conditions.

Although our study sample could be considered representative with respect to age, gender, and number of comorbidities, surveys might be subject to response bias [45]. For example, the characteristics of responders may be different from those of non-responders. Furthermore, respondents may not be aware of the reasons for how they respond due to lack of memory about the phenomenon or even fatigue, and/or the respondents do not feel comfortable providing responses that cast on them a negative light. To ensure validity, the survey used in this study was carefully developed and tested through three steps. The project group developing the survey consisted of researchers with experience in survey methodology and clinical knowledge in RMDs, a representative from a user organization (the Norwegian League Against Rheumatism), as well as persons living with RMDs [49]. We consider it a strength that persons living with RMDs and an expert panel were involved in the development as well as in the piloting of the survey [44].

Regarding reliability, another strength is that the survey was built on validated instruments and scales [21,25], and the list of DS was chosen due to its common use in nutritional studies such as NORKOST 3 and NAFKAM 2018 [22,23]. Furthermore, among the predefined list of 32 DS, the option “other” was also included, allowing the participants to report any other DS not mentioned in the list. A limitation might be that the survey was quite extensive to fill out, which might be an explanation for the relatively low response rate of 8.65%. In addition, as stated in the recent national survey of 2021 in Norway, digital skills are linked to gender, age, level of education, and long-term illness [43]. In line with Arnesen et al. [31], conducting the survey via e-mail and a shorter survey with fewer questions could have ensured a higher response rate. Due to the convenience sampling technique, the generalization of the study findings to the target population should be conducted with a certain level of caution [50]. The study follows the STROBE Statement checklist for reporting cross-sectional studies [51].

## 5. Conclusions

Our study showed that the use of DS was common among a convenience sample of persons living with FMS in Norway and their yearly expenditure on DS was relatively high, and higher than compared with the normal population, thus confirming our first hypothesis. Vitamin D, magnesium, and omega-3 fatty acids were reported to be the most widely used DS. Users of the DS marketed for muscles and/or joints seem to spend more money on DS. Further, confirming our second hypothesis, self-perceived knowledge about DS was higher than actual knowledge within this group of persons with FMS. However, our third hypothesis that predictors for use were higher age, higher education, good self-perceived health, and a better lifestyle was only partly confirmed. The predictors of being DS users were high education, high self-reported knowledge of DS, a trend towards using reliable information sources, but rather low overall knowledge of health claims.

In conclusion, it seems necessary to improve the population’s health literacy in general, as well as develop interventions fostering informed decisions about DS in patients with FMS. We believe the study will generate important knowledge for future interventions such as tailored educational campaigns capable of fostering informed decisions about DS intake among people with FMS.

## Figures and Tables

**Table 1:** Characteristics of the whole sample and the users and non-users of DS.

	Reg (*n*)	Whole Sample (*n* = 504)	Range (Min/Max)	Users of DS (*n* = 430)	Non-Users of DS (*n* = 74)	*p*
Gender, women (*n*) (%)	504	476 (94.4)		407 (94.7)	69 (93.2)	0.63
Age in years, mean (SD)	504	52.7 (10.1)	21–81	52.8 (10.1)	51.8 (10)	0.43
Marital status, living with a partner (*n*) (%)	504	382 (75.8)		324 (75.3)	58 (78.4)	0.57
Education, high (*n*) (%)	504	253 (50.2)		232 (54)	21 (28.4)	0.01 *
Employment, active (*n*) (%)	504	171 (33.9)		145 (33.7)	26 (35.1)	0.81
Health professional background (*n*) (%)	504	132 (26.2)		117 (27.2)	15 (20.3)	0.21
Number of diagnoses, mean (SD)	504	2.2 (1.3)	1–7	2.3 (1.3)	2.1 (1.3)	0.24
Comorbidity, two or more diagnoses (*n*) (%)	504	311 (61.7)		271 (63)	40 (54.1)	0.14
Number of medications, high (*n*) (%)	412	230 (45.6)		201 (56.9)	29 (49.2)	0.27
BMI, kg/m^2^, mean (SD)	499	28.5 (5.4)	16–46.1	28.4 (5.4)	29.2 (5.4)	0.25
Lifestyle factors, health index, mean (SD)	486	3.4 (0.9)	0–5	3.4 (0.9)	3.3 (0.9)	0.38
Self-reported health in general, mean (SD)	504	3.2 (1.1)	1–6	3.2 (1.1)	3.1 (1.1)	0.36
Self-reported health today, mean (SD)	504	50.3 (17.6)	0–90	50.3 (17.7)	50 (17.3)	0.88
Self-reported knowledge of DS, mean (SD)	504	4.2 (1)	1–6	4.3 (0.9)	3.6 (1.3)	0.01 *
Knowledge about health claims, mean (SD)	504	26.9 (6.2)	10–48	26.7 (6.3)	28.5 (5.7)	0.02 *
Sources of information, reliable (*n*) (%)	504	402 (79.8)		351 (81.6)	51 (31.1)	0.01 *

Note: *p*-value implies the significance level based on the chi-squared test for categorical data and the independent samples *t*-test for interval data between the users and non-users of DS (dietary supplements); * *p* < 0.05. Reg (*n*) = number of registered users, % = percentage, SD = standard deviation. For the blank rows, range as a measure was not applicable since the variable represented categorical data. Gender (0 = male and 1 = female), age expressed in years, living with a partner (0 = no and 1 = yes), education (0 = low and 1 = high), employment (0 = not active and 1 = active), health professional background (0 = no and 1 = yes), number of diagnoses, comorbidity (0 = no and 1 = yes), number of medications (0 = low and 1 = high), body mass index (BMI) calculated from a person’s height and weight (18.5–25 = healthy BMI), lifestyle factors as the health index score (from 0 to 5, where a higher number indicates a better lifestyle), self-reported health in general (range, 1–6; high score indicates better health), self-reported health today (range, 0–100; high score indicates better health), self-reported knowledge of DS (range, 1–6; high score indicates better knowledge), knowledge about health claims (range, 9–54; high score indicates better knowledge), sources of information (0 = not reliable and 1 = reliable).

**Table 2:** Frequency (n) and percentage (%) of the use and non-use of various types of DS (registered users = 430).

Types of DS	Group *	Not Used	Last Year	Monthly	Weekly
Fish oil	Group 1	273 (63.5)	28 (6.5)	12 (2.8)	117 (27.2)
Omega-3 fatty acids	Group 1	165 (38.4)	40 (9.3)	11 (2.6)	214 (49.9)
Multivitamin/-mineral	Group 2	227 (52.8)	43 (10.0)	10 (2.3)	150 (34.9)
Vitamin A	Group 3	398 (92.6)	9 (2.1)	2 (0.5)	21 (4.9)
Vitamin D	Group 3	102 (23.7)	44 (10.2)	10 (2.3)	274 (63.7)
Vitamin E	Group 3	389 (90.5)	17 (4.0)	5 (1.2)	19 (4.5)
Vitamin C	Group 3	206 (47.9)	61 (14.2)	34 (7.9)	129 (30)
Vitamin K	Group 3	377 (87.7)	17 (4.0)	1 (0.2)	35 (8.2)
Vitamin B12	Group 3	220 (51.2)	63 (14.7)	14 (3.3)	133 (31)
Folic acid/folate	Group 3	346 (80.5)	30 (7.0)	3 (0.7)	51 (11.9)
Iodine	Group 3	385 (89.5)	15 (3.5)	3 (0.7)	27 (6.3)
Calcium	Group 3	298 (69.3)	31 (7.2)	8 (1.9)	93 (21.7)
Iron	Group 3	337 (78.4)	39 (9.1)	11 (2.6)	43 (9.9)
Zink	Group 3	355 (82.6)	22 (5.1)	4 (0.9)	49 (11.1)
Magnesium	Group 3	151 (35.1)	43 (10.0)	16 (3.7)	220 (51.1)
Selenium	Group 3	386 (89.8)	11 (2.6)	1 (0.2)	32 (7.4)
Chromium	Group 3	380 (88.4)	19 (4.4)	4 (0.9)	27 (6.3)
Probiotics/prebiotics	Group 4	327 (76.0)	49 (11.4)	7 (1.6)	47 (10.9)
Omega 6 fatty acids	Group 4	370 (86.0)	17 (4.0)	2 (0.5)	41 (9.5)
CLA	Group 4	419 (97.4)	6 (1.4)	0 (0.0)	5 (1.2)
Q10	Group 4	406 (94.4)	12 (2.8)	4 (0.9)	8 (1.8)
Garlic	Group 4	308 (71.6)	26 (6.0)	34 (7.9)	62 (14.4)
Ginseng	Group 4	405 (94.2)	12 (2.8)	5 (1.2)	8 (1.9)
Valerian	Group 4	419 (97.4)	10 (2.3)	0 (0.0)	1 (0.2)
Red sunflower/echinacea	Group 4	406 (94.4)	15 (3.5)	7 (1.6)	2 (0.4)
Rhodiola rosea	Group 4	415 (97.6)	6 (1.4)	2 (0.5)	2 (0.4)
St. John’s wort	Group 4	421 (97.9)	5 (1.2)	3 (0.7)	1 (0.2)
Collagen Plus	Group 5	384 (89.3)	18 (4.2)	2 (0.5)	26 (6.0)
Medox	Group 5	417 (97.0)	6 (1.4)	2 (0.5)	5 (1.1)
VitaPro	Group 5	411 (95.6)	9 (2.1)	0 (0.0)	10 (2.3)
Glucosamine	Group 5	409 (95.1)	14 (3.3)	0 (0.0)	7 (1.6)

* The DS users were categorized into five groups: (1) omega-3 fatty acids or fish oil users, (2) multivitamin or -mineral users, (3) single-vitamin or -mineral users, (4) non-vitamin or -mineral users, and (5) users of the DS marketed specifically for RMDs.

**Table 3:** Lifestyle factors in frequency (*n*) and percentage (%) for the sample.

Lifestyle Factors	Registered	Never/Less often than 1 Day	1–4 Days	5–7 Days
Smoking status	*n* = 501	439 (87.1)	5 (1.0)	57 (11.3)
Snuff habits	*n* = 501	474 (94.0)	5 (1.0)	22 (4.4)
Alcohol use	*n* = 500	296 (58.7)	199 (39.5)	5 (1.0)
Physical activity	*n* = 499	45 (8.9)	332 (65.9)	122 (24.2)
Fruits/vegetables	*n* = 498	55 (10.9)	251 (49.8)	192 (38.2)

**Table 4:** Agreement or disagreement with nine health claims illustrated in frequency (n) and percentage (%).

Health Claims	Correct Answers	
1. All adults need to take DS	245 (48.7)	No
2. DS must refer to the effect before distribution in the market	28 (5.6)	No
3. DS must undergo tests to ensure that they are safe before they can be sold on the market	18 (3.6)	No
4. Taking multivitamin/-mineral products prevents diseases in healthy adults	232 (46.1)	No
5. Taking vitamin C supplements regularly prevents colds	196 (38.9)	No
6. Taking an omega-3 supplement or fish oil regularly prevents cardiovascular disease	67 (13.3)	No
7. Taking antioxidant supplements regularly prevents various cancers	262 (52)	No
8. For the elderly, taking vitamin D supplements regularly will reduce the risk of bone fractures	102 (20.3)	No
9. The use of DS may influence the effect of medicines	388 (77)	Yes

**Table 5:** Unadjusted linear regression of the association between estimated money spent on DS in the last year and variables measuring demographics, lifestyle factors, health conditions, knowledge about DS, and subgroups of users.

Independent Variables	Unadjusted Analyses				
	*n*	R^2^	B (95% CI)	β	Sig.
Gender (0 = women, 1 = men)	504	−0.002	−255.48 (−1836.5–1325.6)	−0.015	0.751
Age in years	504	0.001	21.95 (−13.2–57.1)	0.059	0.221
Marital status (no partner = 0, partner = 1)	504	0.007	−847.71 (−1669.3–−26.1)	−0.098	0.043 *
Education (low = 0, high = 1)	504	0.016	939.27 (231.1–1647.5)	0.125	0.009 *
Employment (not active = 0, active = 1)	504	−0.002	84.87 (−667.7–837.4)	0.011	0.825
Health professional background (no = 0, yes = 1)	504	−0.002	−88.08 (−887.5–711.3)	−0.010	0.829
Number of diagnoses	504	−0.001	102.1 (−170.8–377)	0.036	0.460
Number of medications (low = 0, high = 1)	412	0.002	524.92 (−257.8–1307.7)	0.07	0.19
BMI, kg/m^2^	499	0.002	−44.23 (−110.1–21.6)	−0.064	0.187
Lifestyle factors, health index	486	−0.001	−143.75 (−570.7–283.2)	−0.033	0.508
Self-reported health in general	504	−0.002	36.89 (−286.7–360.5)	0.011	0.823
Self-reported health today	504	−0.002	−4.71 (−24.9–15.4)	−0.022	0.646
Self-reported knowledge of DS	504	0.010	465.40 (6936–861.4)	0.111	0.021 *
Knowledge about health claims	504	0.048	−132.97 (−188.2–−77.8)	−0.223	0.001 *
Sources of information, reliable	504	−0.001	−390.52 (−1308.5–527.5)	−0.04	0.404
Omega-3 fatty acids or fish oil users of DS (profile 1)	332	0.010	1010.65 (161.7–1859.6)	0.112	0.020 *
Multivitamin or -mineral users of DS (profile 2)	171	0.003	552.22 (−158.5–1263)	0.074	0.127
Single-vitamin or -mineral users of DS (profile 3)	373	0.001	1132.46 (−640.8–2905.7)	0.061	0.210
Non-vitamin or -mineral users of DS (profile 4)	96	0.008	873.66 (73.5–1673.8)	0.104	0.032 *
Users of the DS marketed for muscles/joints (profile 5)	88	0.036	1908.21 (993.3–2823.1)	0.194	0.001 *

Note: * *p* < 0.05; *n* = number of registered users, R^2^ = adjusted coefficient of determination, B = unstandardized beta, CI = 95% confidence interval, β = standardized beta, sig. = levels of significance (*p*-value). Gender (0 = male and 1 = female), age expressed in years, living with a partner (0 = no and 1 = yes), education (0 = low and 1 = high), employment (0 = not active and 1 = active), health professional background (0 = no and 1 = yes), number of diagnoses, number of medications (0 = low and 1 = high), body mass index (BMI) calculated from a person’s height and weight (18.5–25 = healthy weight), lifestyle factors as the health index score from 0 to 5 where a higher number indicates a better lifestyle, self-reported health in general (range, 1–6; high score indicates better health), self-reported health today (range, 0–100; high score indicates better health), self-reported knowledge of DS (range, 1–6; high score indicates better knowledge), knowledge about health claims (range, 9–54; high score indicates better knowledge), sources of information (0 = not reliable and 1 = reliable). Five user profiles: (1) omega-3 fatty acids or fish oil users, (2) multi-vitamin or -mineral users, (3) single-vitamin or -mineral users, (4) non-vitamin or -mineral users, and (5) users of the DS marketed specifically towards RMDs.

**Table 6:** Adjusted linear regression analysis of the association between the money spent on DS in the last year (dependent variable) and significant independent variables from the unadjusted analyses in addition to age and gender.

Independent Variables	Adjusted Analysis R^2^ = 0.087			
	*n*	B (95% KI)	β	Sig.
Gender (0 = women, 1 = men)	504	−815.65 (−2352.2–721)	−0.049	0.297
Age in years	504	11.81 (−22.7–46.3)	0.032	0.501
Marital status (no partner = 0, partner = 1)	504	−768.69 (−1567.2–29.8)	−0.090	0.059 #
Education (low = 0, high = 1)	504	819.01 (115.8–1522.3)	0.110	0.023 *
Self-reported knowledge of DS	504	263.82 (−128–655.7)	0.063	0.186
Knowledge of health claims	504	−114.01 (−169.5–−58.5)	−0.193	0.001 *
Omega-3 fatty acids or fish oil users of DS (profile 1)	332	552.62 (−271.8–1379.1)	0.062	0.189
Non-vitamin or -mineral users of DS (profile 4)	96	283.41 (−516.6–1083.4)	0.034	0.487
Users of the DS marketed for muscles or joints (profile 5)	88	1224.99 (294–2156)	0.125	0.010 *

Note: * *p* < 0.05; # *p* < 0.1 (statistical trend); *n* = number of registered, R^2^ = adjusted coefficient of determination, B = unstandardized beta, CI = 95% confidence interval, β = standardized beta, sig. = levels of significance (*p*-value). Gender (0 = male and 1 = female), age expressed in years, living with a partner (0 = no and 1 = yes), education (0 = low and 1 = high), self-reported knowledge of DS (range, 1–6; high score indicates better knowledge), knowledge about health claims (range, 9–54; high score indicates better knowledge). User profiles: group 1: omega-3 fatty acids or fish oil users, group 4: non-vitamin or -mineral users, and group 5: users of the DS marketed specifically towards RMDs.

**Table 7:** Unadjusted and adjusted logistic regression models of the strength of relationships between the users and non-users of DS and variables measuring demographics, lifestyle factors, health conditions, and knowledge of DS.

Independent Variables	Unadjusted Analyses			Adjusted Analysis		
	B	OR (95% CI)	Sig.	B	OR (95% CI)	Sig.
Gender (0 = women, 1 = men)	−0.25	0.78 (0.29–2.12)	0.63	−0.21	0.81 (0.27–2.41)	0.71
Age in years	0.01	1.01 (0.99–1.04)	0.43	0.01	1.01 (0.98–1.03)	0.60
Marital status (non-partner = 0, partner = 1)	−0.71	0.84 (0.47–1.53)	0.57			
Education (low = 0, high = 1)	1.08	2.96 (1.72–5.07)	0.001 *	0.88	2.41 (1.36–4.27)	0.003 *
Employment (not active = 0, active = 1)	−0.06	0.94 (0.56–1.58)	0.81			
Health professional background (no = 0, yes = 1)	0.39	1.47 (0.80–2.69)	0.21			
Number of diagnoses	0.12	1.13 (0.92–1.38)	0.28			
Number of medications (low = 0, high = 1)	0.31	1.37 (0.79–2.38)	0.27			
BMI, kg/m^2^	−0.03	0.97 (0.93–1.02)	0.25			
Lifestyle factors, health index	0.13	1.14 (0.85–1.53)	0.38			
Self-reported health in general	0.11	1.12 (0.89–1.40)	0.33			
Self-reported health today	0.01	1.01 (0.99–1.02)	0.87			
Self-reported knowledge of DS	0.70	2.02 (1.59–2.56)	0.001 *	0.59	1.81 (1.40–2.32)	0.001 *
Knowledge about health claims	−0.05	0.95 (0.92–0.99	0.02 *	−0.07	0.94 (0.90–0.98)	0.004 *
Sources of information, reliable	0.70	2.01 (1.16–3.47)	0.01 *	0.52	1.69 (0.92–3.11)	0.09 #

Note: * *p* < 0.05; # *p* < 0.1 (statistical trend). B = unstandardized beta, OR = odds ratio, CI = 95% confidence interval, sig. = levels of significance (*p*-value). Gender (0 = male and 1 = female), age expressed in years, living with a partner (0 = no and 1 = yes), education (0 = low and 1 = high), employment (0 = not active and 1 = active), health professional background (0 = no and 1 = yes), number of diagnoses, number of medications (0 = low and 1 = high), body mass index (BMI) calculated from a person’s height and weight (18.5–25 = healthy weight), lifestyle factors as a health index score from 0 to 5 where a higher number indicates a better lifestyle, self-reported health in general (range, 1–6; high score indicates better health), self-reported health today (range, 0–100; high score indicates better health), self-reported knowledge of DS (range, 1–6; high score indicates better knowledge), knowledge about health claims (range, 9–54; high score indicates better knowledge), sources of information (0 = not reliable and 1 = reliable).

## Data Availability

The authors confirm that the data supporting the findings of this study are presented in the article.
